# Metabolomics Application in Maternal-Fetal Medicine

**DOI:** 10.1155/2013/720514

**Published:** 2013-06-09

**Authors:** Vassilios Fanos, Luigi Atzori, Karina Makarenko, Gian Benedetto Melis, Enrico Ferrazzi

**Affiliations:** ^1^NICU, Puericulture Institute and Neonatal Section, AOU and University of Cagliari, 09124 Cagliari, Italy; ^2^Department of Biomedical Science, University of Cagliari, 09124 Cagliari, Italy; ^3^Department of Woman, Mother and Neonate, Buzzi Hospital, Biomedical and Clinical Sciences School of Medicine, University of Milan, 20122 Milan, Italy; ^4^Department of Obstetrics and Gynecology and Human Reproduction Physiopathology, University of Cagliari, 09124 Cagliari, Italy

## Abstract

Metabolomics in maternal-fetal medicine is still an “embryonic” science. However, there is already an increasing interest in metabolome of normal and complicated pregnancies, and neonatal outcomes. Tissues used for metabolomics interrogations of pregnant women, fetuses and newborns are amniotic fluid, blood, plasma, cord blood, placenta, urine, and vaginal secretions. All published papers highlight the strong correlation between biomarkers found in these tissues and fetal malformations, preterm delivery, premature rupture of membranes, gestational diabetes mellitus, preeclampsia, neonatal asphyxia, and hypoxic-ischemic encephalopathy. The aim of this review is to summarize and comment on original data available in relevant published works in order to emphasize the clinical potential of metabolomics in obstetrics in the immediate future.

## 1. Introduction

Metabolomics (or metabonomics) is the youngest and possibly the most promising among “omics technologies.” It represents a holistic approach to the metabolites that constitute a cell, tissue, or organism [1]. It is able to provide a phenotypic fingerprinting of a cell, tissue, or organism by virtue of its ability to measure multiple metabolites directly from complex biological systems. Any molecule less than 1 kDa in mass can be sorted out by metabolomics technology as a single final product of active/inactive genes in a given condition (genome), its activation (mRNA transcriptome), the setup of enzymatic machineries (proteome), and their actual biological processes.

Metabolomic studies in pregnancy are not a novel concept. Since the 1960s, the role of specific single metabolites in the dynamic interactions among fetus, placenta, and pregnant woman had been reported in various studies [2–6]. However, metabolomics in pregnancy, under present high-through-output technologies, is still an “embryonic” science: in 2009, the first review on this topic had been published based on limited data [1], the state of art situation has not changed much since then except for the fact that recent studies prove again and again the immense possibilities of this technology in the complex field of pregnancy where two partner organisms are interacting for mutual benefit [5, 7–11]. 

## 2. The Metabolomic Approach

The metabolomic approach consists of two sequential phases. The analytical phase is designed to profile all low molecular weight metabolites in a given biological specimen to generate an all-inclusive spectrum. Possible sources of biological tissues that can be exploited in pregnancy are both from the mother (plasma, urine, vaginal fluids, milk), the fetus (amniotic fluid, umbilical cord blood), and the newborn (plasma, urine, placenta, saliva, other fluids). Different technologies might be generally adopted: nuclear magnetic resonance (NMR) spectroscopy, gas chromatography-mass spectrometry (GC-MS), and liquid chromatography-mass spectrometry (LC-MS). The phase of data analysis and interpretation requires highly complex data mining software and an ample database of metabolites to describe the nodes and networking of metabolic pathways [7, 8, 12–27]. In a recent paper, we described in detail these phases in studies performed in pregnant women and neonates [25].

## 3. Metabolomics in Plasma of Pregnant Women 

To the best of our knowledge, metabolomic analysis performed on plasma of pregnant women had been reported in eight studies [2, 7, 8, 18, 26–29] addressing different pregnancy complications. Plasma samples were analyzed with NMR or LC-MS. Main results of these studies are reported in Table 1.

Lower plasma betaine and trimethylamine-N-oxide concentrations [18] were observed in maternal plasma in case of *fetal malformation*. Additional different significant findings were reported for amino acids involved in gluconeogenesis, for cis-aconitate, acetone, 3-hydroxybutyrric, and hypoxanthine. These data suggest that the malformed fetuses demand enhanced gluconeogenesis and tricarboxylic acids cycle (Krebs Cycle), possibly due to hypoxic metabolism. Significant differences were observed also between normal pregnancies and cases which would later develop* gestational diabetes*. Changes in 3-hydroxyisovalerate and 2-hydroxyisobutirrate were observed, suggesting early changes in biotin status and altered aminoacid and/or gut metabolism. Preeclampsia (PE) had been a major focus in metabolomics technologies applied to maternal-fetal medicine. Both Kenny et al. [26] and Odibo et al. [27] focused their studies on predicting preeclampsia. Kenny's work was mainly focused on late preeclampsia of maternogenic origin, as we can argue from the limited prevalence of small for gestational age (SGA) newborns in his cohort, and early and late PE were not objects of a different analysis. Odibo in a slightly smaller sample observed a lower detection rate for early onset preeclampsia only. Unfortunately, the lack of consistent classification of hypertensive disorder in pregnancy as regards the relationship between early onset preeclampsia and late onset preeclampsia and placental vascular damage [17] might impact adversely the correlations observed in the metabolome in cases with and cases without early placental vascular damage and fetal growth restriction (FGR) [9]. 

Very recently Bahado-Singh et al. [7, 8] published 2 papers focusing on early onset and late onset preeclampsia, selected by the calendar based definition of early and late preeclampsia and by evidence of placental damage in early onset preeclampsia. NMR-based metabolomic analysis was performed on first-trimester maternal serum at 11–13 weeks of gestation to assess the possible prediction of late preeclampsia. A parsimonious genetic computing model proved yielded 60% sensitivity at 96.6% specificity for late preeclampsia prediction. A comparison with database of the previous study on early onset preeclampsia proved a strong separation of late-versus early-onset preeclampsia groups. Two metabolites based on the VIP plot were notable for their differences between preeclamptic and normal pregnant women: glycerol and carnitine. Glycerol and carnitine are both important for lipid metabolism and mitochondrial energy productions based on lipids. More significantly carnitine inhibits oxidative stress. All these metabolic pathways are overexpressed in metabolic syndrome and its acute expression in late preeclampsia.

## 4. Metabolomics in Urine of Pregnant Women and Neonates

Table 2 reports the relevant methodology, results, and conclusions of five studies investigation metabolomics findings in urine of pregnant women and neonates [30–34]. In pregnant women with fetuses affected by somatic abnormalities metabolomics findings confirmed enhanced plasmatic concentration of glucogenic amino acids as well as an increase in hypoxanthine concentration and tricarboxylic acid cycle suggesting ATP degradation and fetal stress. 

A possible role of methionine, phenylalanine, histidine, and hexose (possibly glucose) changes in preterm delivery has been suggested.

Inositol phosphoglycan P type (P-IPG) has been linked to insulin resistance, thus making its raise an important marker in Gestational Diabetes Mellitus (GDM) evaluation, fetal growth alterations, and preeclampsia prediction.

Generic markers of *prenatal disorders*, such as preeclampsia, hypoxia, chromosomal disorders, or prediagnostic GDM, are N-methyl-2-pyridone-5-carboxamide and N-methylnicotinamide (products of abnormal nucleotide metabolism) most likely increasing due to the effects of stress.

In a recent paper by Diaz and coworkers [18], maternal plasma and urine were collected and analysed with NMR. Metabolic alterations observed in maternal biofluids in pregnancies with fetal malformations, chromosomal disorders, GDM, PPROM, and preterm delivery suggest potential use of metabolomics in identifying women at risk of prenatal and maternal pregnancy-related disorders. The study by Dessì and coworkers on neonatal urine demonstrated the potential of metabolomics in identifying FGR foetuses and contributing to their clinical management [34] with metabolomics analysis of neonatal urine being another promising field for further investigation [19, 20, 35]. In a preliminary study by our group, a metabolomic approach on maternal urine was used for the diagnosis of labor, suggesting that it is possible to anticipate the timing of delivery [36]. 

## 5. Metabolomics in Amniotic Fluid

Metabolomic analysis of amniotic fluid, mainly determined with NMR and MS (ULPC and LC), has been reported by several studies addressing different pregnancy complications [3, 30, 37–41]. Main results of these studies are presented in Table 3.

Amniotic fluid biomarkers seemed to have the best predictive value for malformed fetuses; in this case, lower glucose and higher free lactate levels indicate that energy production is being conducted preferentially through glycolysis under anaerobic (hypoxic) conditions and a reduced use of mitochondrial respiratory chain pathway (reflected by succinate increase). Due to hypoxia less glucose is available to other tissues and in order to replenish the glucose level an augmented glucose production via gluconeogenesis is observed (with a resulting decrease in gluconeogenic amino acids levels).

Higher glutamine level, glutamine/glutamate ratios, increase in glycoprotein P1, and lower urea level reflect kidney disorders/underdevelopment. Changes in glycine, serine, *α* oxoisovalerate, leucine, and ascorbate suggest a disturbance in amino acid biosynthesis. Amniotic fluid changes described above are characteristic for the whole group of malformations indicating changes in glycolysis, gluconeogenesis, and kidney underdevelopment.

Data regarding GDM (increase of glucose level consistent with previously reported insulin increase; slight reduction of several amino acids, creatinine, acetate, formate, glycerophosphocholine) suggest changes in amino acids biosynthesis, higher demand for protein, and changes in lipid metabolism, and renal function.

A derangement of amino acid metabolism due to observed increase in succinic acid and glutamine concentration and significantly lower concentrations of creatine and creatinine has been suggested for the spina bifida cases analyzed with metabolomics techniques.

The changes observed in the amniotic fluid related to preterm delivery include increase of allantoine (a marker of oxidative stress) and a decrease of myoinositol that promotes fetal lung maturation.

Two recent cross-sectional metabolomics studies reported the importance of the amniotic fluid metabolic signature for preterm labor (PTL) with or without intra-amniotic infection or inflammation (IAI) [3, 41]. Amniotic fluid biomarkers were able to discriminate 3 groups based on the pregnancy outcome [41]: (1) patients with PTL who delivered at term, (2) patients with PTL without IAI who delivered preterm, and (3) patients PTL with IAI who delivered preterm (characterized by an increase in amino acid metabolites). A reduction of carbohydrates in the amniotic fluid was associated with preterm delivery (with or without IAI). Methyladenine and diaminopimelic acid, components of bacterial processes and bacterial wall, respectively, were the most significant biomarkers in this clinical condition.

These studies show the potential of human amniotic fluid metabolome analysis as the basis for the development of rapid tests to differentiate between patients at risk of impending preterm birth (with or without infection/inflammation) and patients who will deliver at term.

## 6. Metabolomics in Placenta

Metabolomic analysis of placenta has been reported in several scientific papers [4–6, 10, 11]. The results of these studies are presented in Table 4.

Dunn and coworkers [4] studied the metabolomic response of the normal and preeclamptic placental tissue to different oxygen levels. Placental tissue from uncomplicated pregnancies cultured in 1% oxygen (hypoxia) showed metabolic similarities to explants from late-onset preeclampsia pregnancies cultured at 6% oxygen (normoxia) thus suggesting an important role of hypoxia in preeclampsia development. Specific metabolic alterations included lipids, glutamate, glutamine, and metabolites related to tryptophan, leukotrienes, and prostaglandins. These metabolic changes were confirmed by Horgan and coworkers [11] that in a similar study compared placental features seen in small for gestational age (SGA) cases versus controls. Placental tissue from both groups was cultured in 1%, 6%, and 20% oxygen. There was a significant difference in more than 500 metabolites between SGA and normal pregnancies at different oxygen tensions, while 49% of metabolites of interest were similar under normoxic conditions suggesting in vivo adjustment to hypoxia by SGA fetus, with a further consideration that normal oxygen level could worsen metabolic dysfunction of the placenta of a SGA fetus. This study reinforces the role of hypoxia and oxidative stress in preeclampsia and SGA. The role of chronic hypoxia was also studied by Tissot van Patot and colleagues [6], who reported the placentas at 3100 m sea level, naturally present higher concentrations of stored energy potential, antioxidants, and lower free amino acid concentrations, with better resistance to hypoxia and less evident metabolic stress response, and their results suggesting adaptation to chronic hypoxia in placenta at high altitude.

## 7. Metabolomics in Vaginal Secretion

A pilot mass spectrometry metabolomic study of women who had a *spontaneous preterm birth* compared to controls was conducted using the uterine cervical length as a selective factor. Noninvasive collection of cervicovaginal secretions from 15 women (women with a short cervix who had a spontaneous preterm birth compared to a control cohort with a short cervix and with a long cervix) was performed, and 17 markers of interest were identified. This particular metabolomic approach may help in the management of women at highrisk for spontaneous preterm birth [16].

## 8. Metabolomics in Cord Blood

Metabolomic analysis of cord blood has been carried out on samples collected from low birth weight (LBW) newborns, very low birth weight (VLBW) newborns, newborns diagnosed with FGR, SGA during pregnancy, newborns with asphyxia, and hypoxic ischemic encephalopathy. The results were reported in five studies [29, 42–45]. In the paper by Ivorra and coworkers [42] seven metabolites were able to discriminate a specific LBW metabolome, with a significant positive correlation between birth weight and proline, glutamine, free choline and negative correlation with citrulline and phenylalanine levels. These findings demonstrate the differences in the anabolic rate of the LBW newborns and the possible consequences that these metabolites' modifications can exercise on the newborn's health (e.g., maternal choline and its metabolite betaine supply modifying fetal histone and DNA methylation). It is also noteworthy that these changes were observed in the newborns only and not in their mothers, suggesting possible involvement of impaired placental transport of amino acids. Metabolic findings described above reveal the potential of metabolomics in identifying the population at risk. Tea and coworkers [43], analysing the cord blood from VLBW infants, demonstrated that a number of metabolites (glucose, acetate, lipids, pyruvate, glutamine, valine, threonine,) vary depending on gestational age at delivery. In the study by Favretto and coworkers [44], FGR presented changes in 22 metabolites with the most evident modifications in the following aminoacids composition: phenylalanine, tryptophan, and glutamate with 91–100% sensitivity and 85–89% specificity. Cut-off values for phenylalanine and tryptophan were identified. This study demonstrates that metabolomics is a promising tool for identification of FGR fetuses.

Horgan and coworkers [29] observed that 19 metabolites (including sphingolipids, phospholipids, carnitines) differentiate SGA from controls, providing a base for a valid screening test for presymptomatic SGA or FGR. Early identification of cases at risk would help to develop adequate clinical management of patients to avoid serious complications. Another study that confirms one more time the importance of metabolomic analysis for the future of the medicine, in this case producing a robust predictive model for detecting encephalopathy in the newborns at 24 hours of life, in order to be able to provide the best timely treatment for the young patient is that of Walsh and coworkers [45]. This study found 29 metabolites among amino acids, acylcarnitines and glycerophospholipids to be significantly different in neonates with hypoxic ischaemic encephalopathy (HIE) versus controls or asphyxia versus controls groups, with 5 metabolites (2 acylcarnitines, 1 glycerophospholipid and 2 aminoacids) clearly delineating the severity of asphyxia and the degree of HIE. 

All these data suggest that metabolomics is a noninvasive promising novel way of investigation, with the goal of understanding better the pathogenesis of different diseases, developing screening tests and helping in clinical management of the patients. 

## 9. Conclusions

Metabolomics appears to be one of the most promising novel tools in obstetrics, among other important “omics”. A recently published paper was entitled “Can biofluids metabolic profiling help us to improve health care during pregnancy?” [46]. The answer is “probably yes” if all these preliminary data on the potential usefulness of biofluids metabolomics in perinatal disorders are further investigated, single diseases are targeted and results are confirmed. 

Through the metabolome we can observe the enormous and complex interactions between mother, placenta, and fetus. This high throughput technology might allow us to identify key role nodes for prediction, diagnosis, and monitoring of different obstetrics conditions. 

## Figures and Tables

**Table 1:** Methodology, findings, and main conclusions derived from metabolomics studies on maternal plasma.

Population	Gestational age at examination	Metabolomic analysis	Main differences in abnormal pregnancies	Significance/take-home messages	Reference	Author, year
20 normal pregnancies	Matched					
27 fetal malformations	At diagnosis		Gluconeogenetic aminoacids, *cis*-aconitate, acetone, 3-hydroxibutirric, hypoxanthine	Pregnant women with malformed fetus showed enhanced glucogenesis and tricarboxylic acids cycle		
23 chromosomal disorders	At diagnosis	NMR			[18]	Diaz et al., 2011
14 prediagnostic GDM	Before positive OGTT		3-Hydroxyisovalerate, 2-hydroxyisobutirrate	Pregnant women who later develop GDM showed early changes in biotin status and altered aminoacid and/or gut metabolism		
18 pPROM	At diagnosis		No alterations			

11 normotensive pregnancies versus 11 preeclamptic pregnancies	n.r.	NMR	Hystidine, tyrosine, phenylalanine	Diagnosis of preeclampsia was possible by metabolomics	[28]	Turner et al., 2008

60 cases of PE versus matched controls	15 ± 1 weeks	UPLC-MS	40 metabolites (detection rate 71%, 5% false positive)	Metabolomics might predict preeclampsia, early and late PE not analysed separately	[26]	Kenny et al., 2010

41 cases of PE versus controls	First trimester	LS/MS	40 acylcarnitine species and 32 aminoacids (AUC-ROC = 0.85)	Metabolomics might predict early onset PE	[27]	Odibo et al., 2011

30 cases of early PE versus 60 controls	First trimester	NMR	Citrate, glycerol, hydroxyisovalerate, and methionine and uterine Doppler abnormal PI (detection rate of 82.6%, at an FPR of 1.6%)	Metabolomics might predict early onset PE	[7]	Bahado-Singh et al., 2012

30 cases of late PE versus 119 controls	First trimester	NMR	17 metabolites	Metabolomics might predict late onset PE	[8]	Bahado-Singh et al., 2013

Pregnant women who subsequently delivered an SGA baby	First trimester	UPLC-MS	19 metabolites (OR = 44; AUC-ROC = 0.90)	Metabolomics might predict SGA	[29]	Horgan et al., 2011

GDM: gestational diabetes mellitus; pPROM: preterm premature rupture of membranes; OGTT: oral glucose tolerance test; NMR: nuclear magnetic resonance spectroscopy; PE: preeclampsia; UPLC-MS: ultra performance liquid chromatography-mass spectrometry; LS/MS: liquid chromatography-mass spectrometry; SGA: small for gestational age.

**Table 2:** Methodology, findings, and main conclusions derived from metabolomics studies on maternal and neonatal urine.

Population study	Gestational age at examination	Metabolomic analysis	Main results	Significance/take-home messages	Reference	Author, year
48 GDM cases versus 23 controls	Third trimester	MS	Increased urinary excretion of P-IPG, positive correlation with blood glucose	Metabolomic identification of P-IPG is a potential marker of insulin resistance, may predict fetal growth alterations in GDM patients	[33]	Scioscia et al., 2007

9 PE cases versus 84 controls	First trimester	Elisa-based assay	Rapid raise of P-IPG (sensitivity and specificity of 88.9% and 62.7%, resp.)	Metabolomics by multiple assessment of samples might predict preeclampsia	[32]	Paine et al., 2010

3083 pregnancies positive to the screening of Smith-Lemli-Opitz syndrome	Second trimester	GC/MS	16*α*-OH-DHEA, urine total estriol. Fetal steroid sulfatase deficiency 98% detection rate, 95% CI 92–99	Metabolomics analysis of Maternal urine steroids is effective in detecting STSD. Possible use for diagnosing ADHD and CGDS (common in association with STSD)	[31]	Marcos et al., 2009

75 pregnancies (13 healthy)				Potential of the tandem use of MS and NMR for metabolomics studies of urine and amniotic fluid in pregnant women		
13 fetal malformations			Hippurate, amino acids			
20 predisposition to GDM	Second trimester	UPLC-MS NMR	No relevant changes		[30]	Graça et al., 2012
6 preterm delivery			Methionine, phenylalanine, hystidine, hexose (possibly glucose)			

26 preterm FGR versus 30 preterm appropriate for gestational age	Neonates	^1^H NMR	Myoinositol, sarcosine, creatine, and creatinine	Metabolomics might identify FGR and contribute to the clinical management of FGR neonates	[34]	Dessì et al., 2011

GDM: gestational diabetes mellitus; MS: mass spectrometry; P-IPG: inositol phosphoglycan P type; PE: preeclampsia; GC/MS: gas chromatography-mass spectrometry; STSD: steroid sulfatase deficiency; ADHD: attention deficit-hyperactivity disorder; CGDS: contiguous gene deletion syndrome; UPLC-MS: ultra performance liquid chromatography-mass spectrometry; NMR: nuclear magnetic resonance spectroscopy; ^1^H NMR: proton nuclear magnetic resonance spectroscopy; FGR: fetal growth restriction.

**Table 3:** Methodology, findings, and main conclusions derived from metabolomics studies on amniotic fluid.

Population study	Gestational age at examination	Metabolomic analysis	Main results	Significance/take-home messages	Reference	Author, year
14 pregnancies with spina bifida fetuses versus 18 controls	Second and third trimester (at amniocentesis and cesarean section)	^1^H NMR	Alterations in the concentrations of succinic acid, glutamine, creatine, creatinine	Metabolomics can point out derangements in aminoacids metabolism in fetuses with spina bifida and help to diagnose it	[40]	Groenen et al., 2004

Healthy pregnancies	Second trimester (16-17 ws)	RP-LC, MS ^1^H NMR	60 metabolites	Extended metabolite database for detecting biomarkers of pregnancy disorders	[39]	Graça et al., 2008

51 healthy pregnancies versus 12 fetal malformations	second trimester (15–24 ws)	NMR	Glucose, free lactate levels, succinate, gluconeogenic aminoacids, glutamine, glycine, urea, glutamine/glutamate ratios	Metabolomics may help detecting malformed fetuses (due to the changes in glycolysis and gluconeogenesis, and their kidney underdevelopment)	[38]	Graça et al., 2009

82 normal pregnancies 27 fetal malformation			22 metabolites, inclusion of new metabolites: ascorbate, *α* oxoisovalerate, creatinine, isoleucine, serine, threonine	Metabolomics might be able to identify biomarkers of prenatal disorders		
27 prediagnostic GDM	Second trimester	^1^H NMR	Glucose, amino acids, organic acids, creatinine, glycerophosphocholine (GPC)		[37]	Graça et al., 2010
12 preterm delivery			Allantoine, alanine, citrate, myoinositol			
34 PROM 10 chromosomopathies			Minor metabolic profile changes no relevant changes			

2 studies including 16 + 40 PTL → at term 19 + 33 PTL without IAI 20 + 40 PTL with IAI	Second and third trimester (22–35 ws)	LC/MS GC/MS	Carbohydrates, amino acids, presence of xenobiotic compounds (salicylamide, bacterial products) Classification of patients at risk for PTL with 88.5% accuracy	Metabolic profiling of amniotic fluid can be used to assess the risk of preterm delivery with or without IAI	[41]	Romero et al., 2010

36 controls 29 fetal malformations 37 prediagnostic GDM 34 preterm delivery	Second trimester	UPLC-MS NMR	New results: changes in pyroglutamate, hippurate No relevant changes	Potential of the tandem use of MS and NMR for metabolomics studies of urine and amniotic fluid in pregnant women	[30]	Graça et al., 2012

^1^H NMR: proton nuclear magnetic resonance spectroscopy; RP-LC: reverse phase liquid chromatography; MS: mass spectrometry; GDM: gestational diabetes mellitus; PROM: premature rupture of membranes; PTL: preterm labor; IAI: intraamniotic infection/inflammation; LC/MS: liquid chromatography/mass spectrometry; GC/MS: gas chromatography/mass spectrometry; UPLC-MS: ultra performance liquid chromatography-mass spectrometry; NMR: nuclear magnetic resonance spectroscopy.

**Table 4:** Methodology, findings, and main conclusions derived from metabolomics studies on placenta tissues.

Population study	Material	Metabolomic analysis	Main results	Significance/take-home messages	Reference	Author, year
9 SGA cases versus 8 controls	Placenta villous explants	UPLC-MS	574 metabolites were significantly different between SGA and controls; 49% of metabolites of interest were the same for SGA explant cultured under hypoxic conditions and controls cultured under normoxic conditions Changes in phospholipids, essential amino acids (tryptophan, methionine, and phenylalanine) concentrations	Metabolomics might predict SGA	[11]	Horgan et al., 2010

11 uncomplicated term pregnancies	Placenta villous explants	GC-MS	Cultured in 1%, 6%, and 20% oxygen. Differences in 2-deoxyribose, threitol/erythritol, hexadecanoic acid	Metabolomics can be applied to placenta studies and could help in detecting hypoxia	[10]	Heazell et al., 2008

6 cases of preeclampsia versus 6 controls	Villous trophoblast	UPLC-MS	47 metabolites in preeclampsia-derived media cultured under normoxic conditions showed similarities to that of uncomplicated pregnancies cultured under hypoxic conditions. Alterations in glutamate and glutamine, leukotrienes and prostaglandins, kynurenine metabolism	Metabolomics might predict preeclampsia of placental origin developed due to hypoxia	[4]	Dunn et al., 2009

8 cases of labor/Cesarean section at 3100 m versus 8 controls with labor/Cesarean section delivery at sea level	Placenta	^1^H NMR, ^31^P NMR	At sea level: metabolic markers of oxidative stress, increased glycolysis, elevated cholesterol, and free amino acids. At 3100 m: metabolic profiles with adaptation to chronic hypoxia, decreased reliance on anaerobic glycolysis; presence of concentrations of stored energy potential (phosphocreatine), antioxidants (taurine, inositol), and low free amino acid concentrations	Metabolomics might help identify subjects under hypoxic stress (chronic hypoxic preconditioning state versus acute ischemic/hypoxic insult)	[6]	Tissot van Patot et al., 2010

SGA: small for gestational age; UPLC-MS: ultra performance liquid chromatography-mass spectrometry; GC-MS: gas chromatography-mass SPECTROMETRY; ^1^H NMR: proton nuclear magnetic resonance spectroscopy; ^31^P NMR: phosphorus nuclear magnetic resonance spectroscopy.
